# A Rare Case of Granulicatella adiacens Gallbladder Abscess Associated With Gallbladder Adenocarcinoma: Diagnostic and Therapeutic Challenges

**DOI:** 10.7759/cureus.67182

**Published:** 2024-08-19

**Authors:** Heather L Mateja, Ruben Neris, Pablo Giuseppucci

**Affiliations:** 1 General Surgery, American University of Antigua, Osbourn, ATG; 2 General Surgery, Western Reserve Health Education, Warren, USA

**Keywords:** intraoperative esophagogastroduodenoscopy, gallbladder abscess, abscess, lymphoma, cholecystectomy, gallbladder adenocarcinoma, nutritionally variant streptococci, granulicatella adiacens

## Abstract

*Granulicatella adiacens*, a nutritionally variant streptococcus, is part of the normal oral, gastrointestinal, and urogenital flora. It is associated with bacteremia, infectious endocarditis, and, rarely, bone and joint infections. *G. adiacens* infections also tend to have high mortality due to diagnostic challenges and antibiotic resistance. Few case reports have documented its role in abscess formation. Here, we report the first known case of *G. adiacens* causing a gallbladder abscess in a patient with gallbladder carcinoma (GBC), a rare but aggressive cancer. Enhanced awareness and improved diagnostic methods are needed to manage such infections and understand their underlying mechanisms, particularly in immunocompromised patients with malignancies.

## Introduction

*Granulicatella adiacens* is a nutritionally variant streptococci (NVS) species that is part of the normal oral, gastrointestinal, and urogenital flora [1]. Infections caused by *G. adiacens* are rare but have been reported in various clinical settings, including endocarditis, bacteremia, osteomyelitis, and septic arthritis [2-4]. There are few case reports of *G. adiacens* as a cause for abscess formation, with three recent case reports from 2018, 2021, and 2023 describing two subcutaneous abscesses, lung abscess, and liver abscess caused by this bacterium, respectively [1,5,6]. It has been suggested that *G. adiacens* is underreported and may often be the cause of culture-negative disease as previous attempts to culture it have been difficult due to this organism being slow-growing and fastidious, requiring specialized medium and technique [1-3]. Thus, as culture and identification techniques become more advanced, *G. adiacens* may be implicated in a growing number of cases.

Additionally, there have been attempts to correlate gallbladder carcinoma (GBC) with the gut microbiota, suggesting that its imbalance may lead to a significant inflammatory response that does not cause, but may promote the progression of GBC [7]. GBC is a relatively rare cancer, although the most common among biliary tract malignancies [7,8]. The prognosis is generally poor, with a five-year survival rate estimated at 5% [8,9]. This is partly attributed to this cancer being difficult to identify preoperatively, often being found incidentally during a cholecystectomy [8,9]. In this case report, we describe a patient suspected of having GBC undergoing cholecystectomy and intraoperatively identifying a gallbladder abscess with a positive culture for *G. adiacens*. This is the first known report identifying this organism in a gallbladder abscess also associated with GBC.

## Case presentation

 Our patient is an 88-year-old female with a past medical history of recurrent non-Hodgkin lymphoma, chronic obstructive pulmonary disease, multiple deep vein thromboses currently on Xarelto®, hypertension, gastrointestinal reflux disease, rheumatoid arthritis, and hypercholesterolemia, who presented to the emergency department in late May 2024 for abdominal pain. She reported experiencing right upper quadrant abdominal pain for the past several weeks, moderate in intensity, constant in frequency, and associated with nausea. The pain was worse with food consumption, but she had no changes in bowel movements. She denied fever, chills, weight changes, or yellowing of the skin.

In October 2023, she was diagnosed with recurrent right breast lymphoma. She was seen by the oncologist and placed on chemotherapy. Her last round of chemotherapy finished in April 2024. However, her most recent positron emission tomography (PET) scan demonstrated high uptake by the gallbladder concerning for possible malignancy. She was then referred to general surgery for further recommendations. She was seen in the office and was recommended to have a right upper quadrant ultrasound (US) and a hepatobiliary iminodiacetic acid (HIDA) scan. 

**Figure 1:**
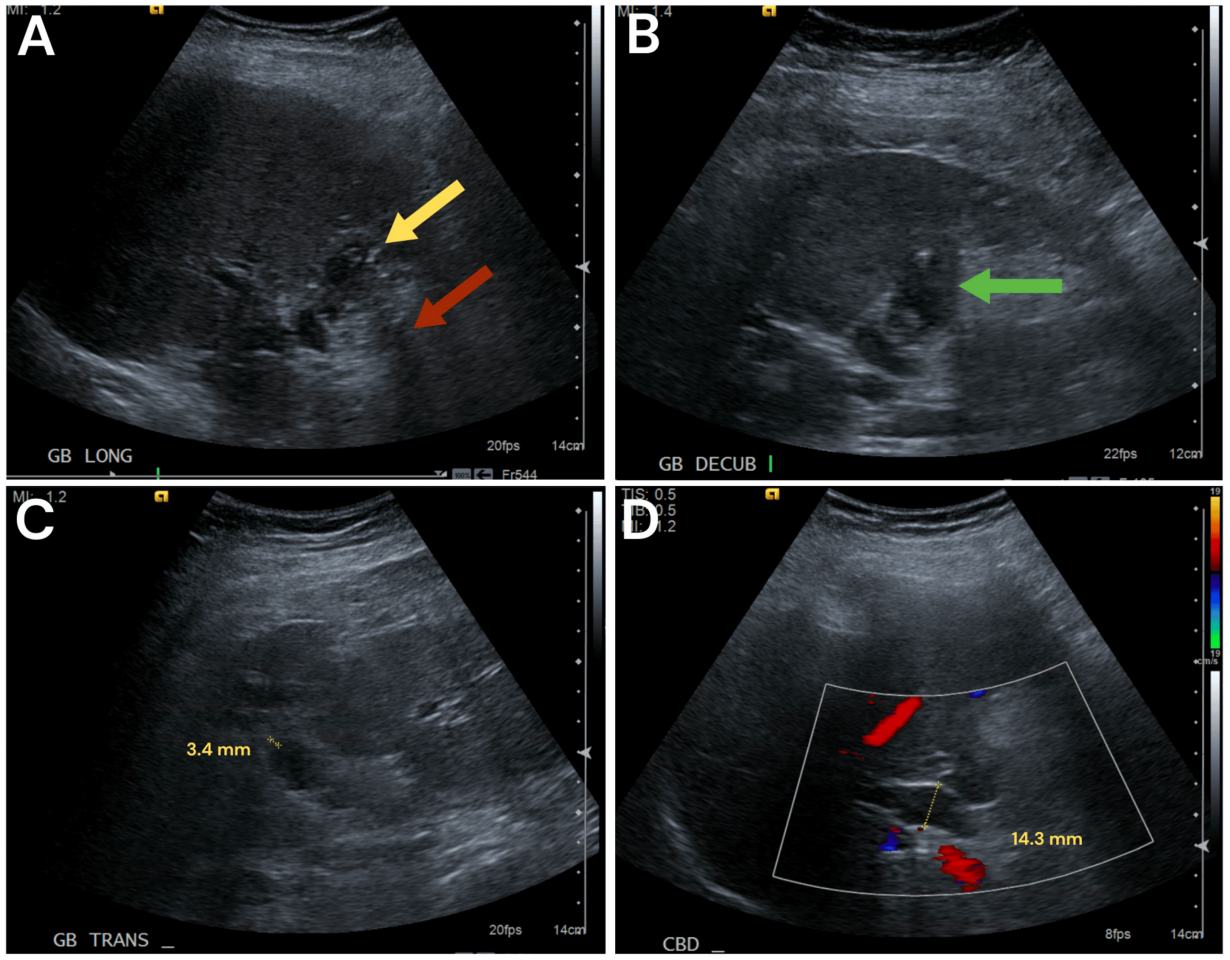
Right upper quadrant ultrasound images (a) Multiple intrinsic echogenic foci (yellow arrow) with acoustic shadowing (red arrow). Ringdown artifact from the margins of the gallbladder. (b) The gallbladder lumen is relatively contracted. No pericholecystic fluid. (c) The gallbladder wall thickness is 3.4 mm, and echogenicity is associated with adenomyomatosis. (d) Common hepatic duct 14.3 mm in anteroposterior (AP) dimension. Intrahepatic bile ducts are not significantly dilated.

**Figure 2:**
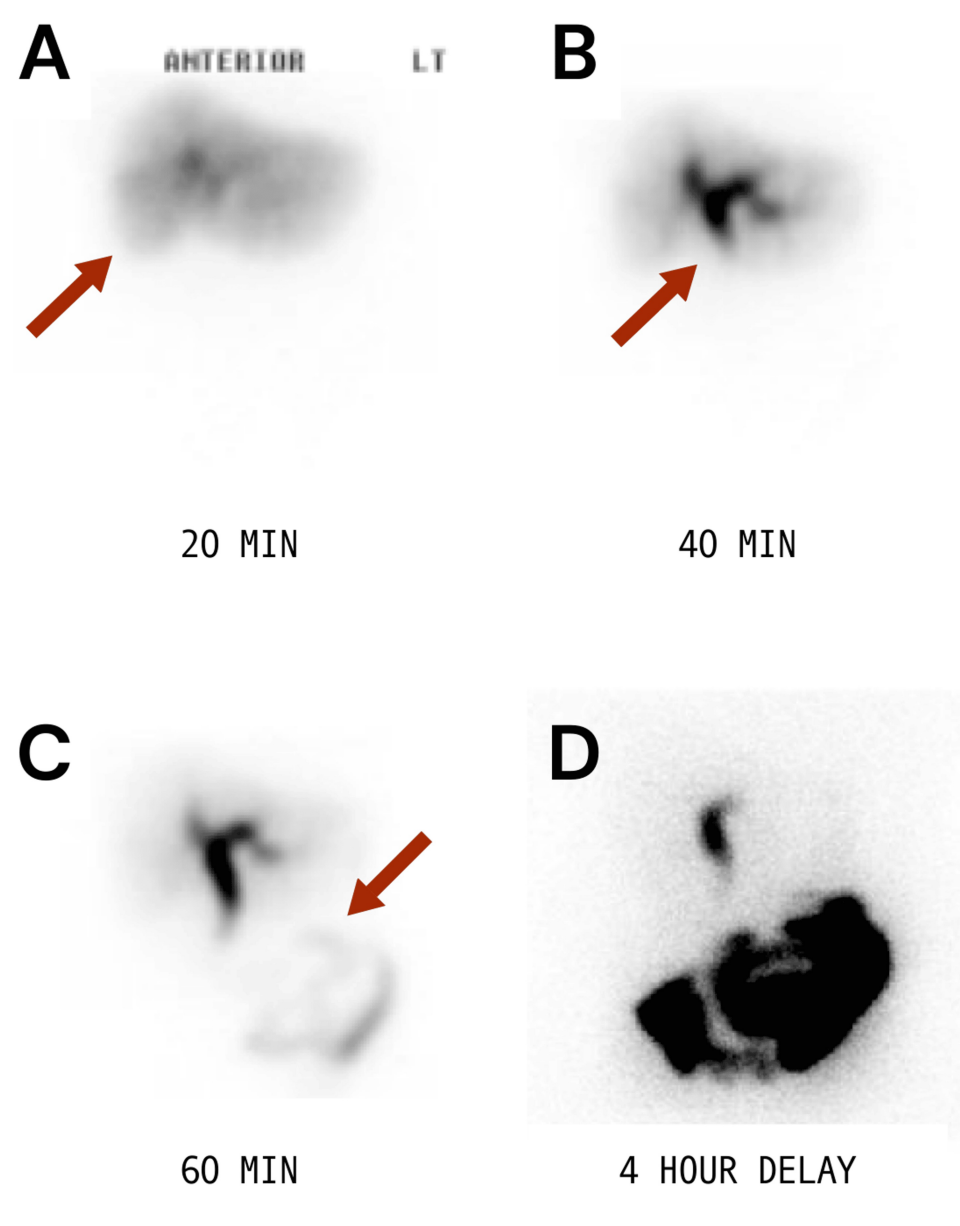
Nuclear hepatobiliary study after intravenous injection 5.03 mCi of technetium 99m mebrofenin (a) Uniform uptake by the liver parenchyma. (b) Bile ducts were visualized at 20 minutes post-injection. (c) Uptake within the bowels, initial activity was at about 45 minutes. (d) Imaging continued for four hours post-injection. The gallbladder is not visualized consistent with cystic duct obstruction.

**Figure 3:**
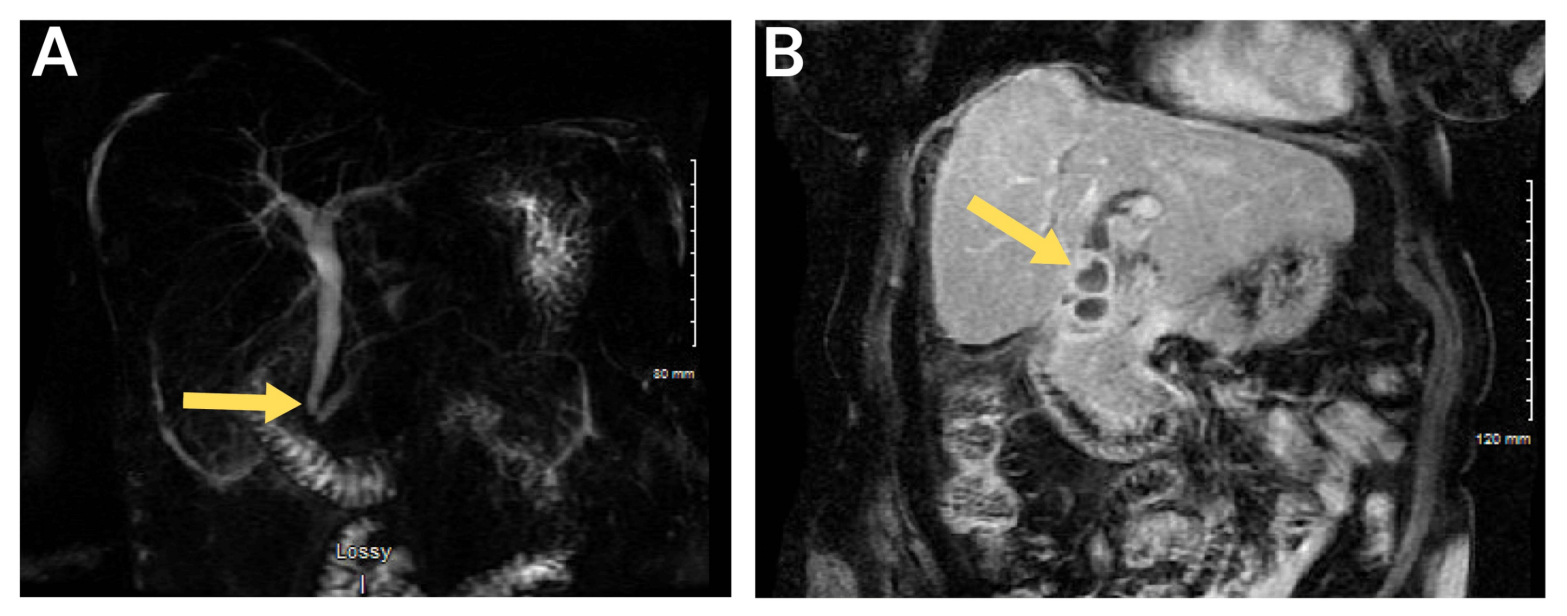
MRCP findings MRCP: Magnetic resonance cholangiopancreatography (a) Mild intrahepatic bile duct dilation with dilated common hepatic duct and common bile duct down to the level of the ampulla. The pancreatic duct was dilated in the pancreatic head. A double duct sign (yellow arrow) was present; however, there was no evidence of a mass in the pancreatic head or ampulla. (b) The gallbladder (yellow arrow) is very irregular in shape, with a thick enhancing wall. There appeared to be some sludge within the gallbladder. A fat plane between the gallbladder and the liver could not be identified. The surrounding inflammation extended down to the duodenal bulb with a trace amount of free fluid around the liver.

US demonstrated cholelithiasis, possible adenomyomatosis, and a common hepatic duct diameter of 1.4 cm (Figure 1). HIDA scan was positive for cystic duct obstruction (Figure 2). The patient was going to follow up in the office, but her symptoms increased in severity, prompting her to go to the emergency department. On arrival, her vitals were within normal limits. White blood cell (WBC) count was mildly elevated at 11.9x10^3^/mL. Liver function tests were within normal limits. It was decided to get additional imaging via magnetic resonance cholangiopancreatography (MRCP) to further evaluate the gallbladder anatomy and biliary tree (Figure 3).

The MRCP was concerning for chronic cholecystitis with extrahepatic ductal dilation, but no clear indication of GBC. The decision was made by the patient and her family to return to the hospital in two days for robotic-assisted laparoscopic cholecystectomy with core needle liver biopsies to assess for malignancy.

**Figure 4:**
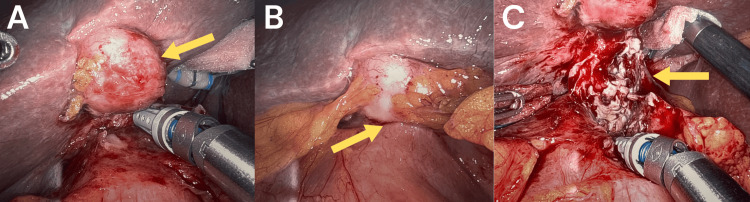
Intraoperative images (a) Indurated and hard gallbladder without an obvious mass. (b) Attachment of the second portion of the duodenum to the fundus of the gallbladder. (c) Abscess cavity at the top of the duodenum.

Upon entering the abdomen, the omentum was safely pulled off the gallbladder and duodenum and retracted caudally. The gallbladder was noted to be indurated and hard without an obvious mass (Figure 4a). The duodenum was noted to be inflamed and erythematous in the second portion and attached to the fundus of the gallbladder (Figure 4b). The dissection was extremely difficult, but carefully, the fundus of the gallbladder was dissected off the duodenum and an abscess cavity was encountered. This was suctioned and the purulent fluid was sent to the laboratory for further analysis. The dissection of the gallbladder off the duodenum was continued, and the fundus was noted to be extremely friable with associated purulent drainage. Finally, the duodenum was successfully detached from the gallbladder and a two-centimeter abscess cavity was visualized at the top of the second portion of the duodenum (Figure 4c).

Given the suspicion of a possible chole-duodenal fistula, the decision was made to perform an esophagogastroduodenoscopy (EGD) intra-operatively. There was no evidence of bubbles, purulent fluid, bleeding, or enterotomy noted. Since the duodenum was intact, the dissection was continued cephalad to the fundus, pushing it above the dome of the liver to obtain better visualization. The remainder of the cholecystectomy proceeded without complication including identification and ligation of the cystic duct and cystic artery. Two biopsies of the liver were taken percutaneously at segments four and five. All biopsies and the specimen were sent to pathology. A Jackson-Pratt (JP) drain was placed near the area of the abscess.

The gallbladder was noted to have moderate to poorly differentiated invasive adenocarcinoma, intestinal type, with infiltration of the gallbladder wall through the entire thickness into the capsule of the liver without involvement of liver parenchyma in the gallbladder bed, extensive necrosis of the tumor with abscess formation, and no lymphovascular permeation of the tumor. Pathologic stage classification based on the American Joint Committee on Cancer (AJCC) eighth edition was pT2b N0 M0. The tumor cells were positive for CK20 and CDX2 but negative for CK7. There was also evidence of acute chronic cholecystitis with cholelithiasis. Both liver segments were unremarkable and negative for malignancy or cirrhosis. However, the serosal margin and cystic duct and margin were involved in invasive carcinoma. Microbiology returned *Granulicatella adiacens* from the abscess culture which, to our knowledge, has not previously been reported as a cause of intra-abdominal gallbladder abscess formation.

The patient received cefazolin and metronidazole pre-operatively as well as ceftriaxone given while remaining in the hospital. She recovered well post-operatively and was discharged the following day with return of bowel function, toleration of diet, and minimal serosanguinous output from the JP drain, which was subsequently removed. 

She returned to the office for a follow-up two weeks post-operatively, at which time she reported no continued signs of infection and no post-operative pain. The patient decided to not pursue chemotherapy or other therapeutic options for the gallbladder adenocarcinoma after a lengthy discussion with her family.

## Discussion

*Granulicatella adiacens* and other NVS infections tend to have a high mortality rate, reported around 9.0%, due to unreliable diagnosis and inappropriate coverage of these organisms [3,10]. Different organizations recommend different antibiotic regimens to cover NVS based on the affected system [3,4,11]. The etiology of the abscess should be ascertained along with antibiotic susceptibilities for the organism [6,10]. Often, a two-combination antibiotic is recommended, particularly with the treatment of infectious endocarditis and disseminated infections [3,11]. *G. adiacens *is usually susceptible to beta-lactam antibiotics with increasing resistance to penicillins [3,5]. Left untreated, these infections have a high complication rate, often leading to bacteremia [11]. Therefore, it is prudent to remain suspicious of culture-negative samples, particularly in the case of intra-abdominal abscess, until more effective culture strategies can be implemented. In our case, the purulent sample from the abscess detected *G. adiacens, *which was treated with ceftriaxone for two days while she remained hospitalized post-procedure without further infectious complications. Since the entirety of the abscess cavity was able to be resected along with the gallbladder and blood cultures were negative for bacteria, long-term antibiotic therapy was not warranted in our case.

Interestingly, the abscess is associated with a concurrent gallbladder adenocarcinoma. While *G. adiacens* is part of the gastrointestinal commensal flora, it remains unclear how the abscess formed, as the EGD ruled out duodenal perforation or fistula [12]. A similar case report also describes the formation of a pyogenic liver abscess due to *G. adiacens* without a direct connection to an area of colonization [6]. It was noted that liver abscesses may form via biliary dissemination in about 40% of cases, which could also explain the formation of a gallbladder abscess in our case [6]. Additionally, in a case of lung abscess formation secondary to lung cancer with *G. adiacens* identified as one of the main pathogens, it is theorized that the tumor itself creates an immunocompromised state facilitating abscess formation from anaerobic species invading oxygen-poor tissue [5]. The implication of this case report, similar to ours, is that identification of *G. adiacens* or other NVS may indicate a serious underlying ailment that may require further investigation in lieu of an identifiable colonization source. This is further exemplified by a rare gallbladder carcinosarcoma that was identified during a workup for a hepatic abscess obscuring the tumor [13]. Gallbladder malignancy is a known risk factor for gallbladder perforation, which can then lead to the dissemination of bacteria [14]. The aforementioned case reports support the pathogenesis of hepatobiliary abscess formation in patients with underlying malignancy.

Considering the information above, there is still the question of how best to manage these patients. As mentioned previously, the overall survival rate for patients with GBC is poor [8,9]. The most important prognostic factor is the extent of the resection, with simple cholecystectomy not being sufficient for T2/T3 tumors [15]. Adjuvant chemotherapy also does not appear to have additional survival benefits, although effective regimens have not yet been established [16]. The standard therapy, if GBC can be identified preoperatively, is radical cholecystectomy with liver wedge resection and lymphadenectomy of the hepatoduodenal ligament in cases of T2 and higher tumors [8,9]. As we did not have the capability to send frozen sections during the operation, and it was unclear if our patient had GBC, it was decided to proceed with simple cholecystectomy and liver biopsies to assess for malignancy, then reassess the patient’s desire for more aggressive treatment.

## Conclusions

Gallbladder carcinoma is a relatively rare cancer with a devastating prognosis. Due to the creation of an immunodeficient microenvironment, this malignancy can lead to gallbladder perforation and subsequent abscess formation. Our patient is an 88-year-old female who presented for a workup of possible gallbladder carcinoma with an intraoperative finding of a gallbladder abscess colonized by *Granulicatella adiacens*, an uncommon bacterium that is notoriously difficult to culture. *G. adiacens* is usually implicated in infectious endocarditis, with very few instances of it causing abscess formation. More information about the etiology of this bacterium and its mechanism of dissemination is needed to fully understand its ability to induce intra-abdominal abscess formation. Being able to appropriately and efficiently culture this organism is essential to prevent the devastating complications that can arise with insufficient coverage.
